# The impact of prolonged air pollution exposure on urinary tract and male reproductive system infections: insights from a large-scale prospective cohort study

**DOI:** 10.1186/s12889-025-24660-6

**Published:** 2025-10-08

**Authors:** Luyao Chen, Lewei Guan, Fuchun Zheng, Yuyang Yuan, Sheng Li, Situ Xiong, Yue Gao, Bin Fu, Lizhi Zhou

**Affiliations:** 1 Department of Urology, Jiangxi Provincial Key Laboratory of Urinary System Diseases, The First Affiliated Hospital , Nanchang University, Nanchang, Jiangxi China; 2 Postdoctoral Innovation Practice Base, The First Affiliated Hospital, Jiangxi Medical College, Nanchang University, Nanchang, 330006 People’s Republic of China; 3 Huankui Academy, Nanchang University, Nanchang, 330000 China; 4 Division of Nephrology and Hypertension, Mayo Clinic, Rochester, MN USA

**Keywords:** Air pollution, UK biobank, Urinary tract infections

## Abstract

**Objective:**

To assess the link between long-term air pollution exposure and the risk of urinary tract infections (UTIs) and male reproductive system infections (MRSIs), which significantly impact incidence and quality of life.

**Methods:**

This prospective cohort study included 375,833 participants from the UK Biobank. We used a Cox proportional hazards model, adjusting for covariates, to evaluate the association between exposure to air pollutants (PM_2.5_, PM_coarse_, PM_10_, NO_2_, and NO_x_) and the risk of UTIs and MRSIs. Nonlinear exposure-response relationships were assessed with restricted cubic splines, and subgroup analyses were conducted to examine potential effect modifiers.

**Results:**

Over a median follow-up of 12.4 years, 26,279 new UTIs and 3,545 MRSI cases were detected. It was found that there were significant connections between the likelihood of developing UTIs and being exposed to PM_2.5_ (hazard ratios (HRs): 1.43, 95% confidence interval (CI): 1.34–1.53), PM_coarse_ (HR: 1.17, 95% CI: 1.08–1.27), PM_10_ (HR: 1.24, 95% CI: 1.15–1.34), NO_2_ (HR: 1.10, 95% CI: 1.08–1.12), and NO_x_ (HR: 1.09, 95% CI: 1.07–1.11). Similar associations were found for MRSIs. Restricted cubic spline analyses revealed non-linear exposure-response relationships for all pollutants. The strength of these connections was validated through sensitivity analyses.

**Conclusions:**

Long-term exposure to air pollutants is associated with a higher risk of UTIs and MRSIs, emphasizing the urgent need to improve air quality to mitigate these health risks.

**Supplementary Information:**

The online version contains supplementary material available at 10.1186/s12889-025-24660-6.

## Introduction

Air pollution is a significant global environmental threat, with growing evidence demonstrating that its health impacts affect much more than just the respiratory system, including cardiovascular, metabolic, and urogenital systems [1–7]. In parallel, urogenital infections—particularly urinary tract infections (UTIs) and male reproductive system infections (MRSIs), such as prostatitis and orchiepididymitis—represent a persistent and substantial public health burden due to their widespread prevalence, considerable morbidity, and associated economic costs [8–12]. Although these two areas of concern have traditionally been investigated separately, increasing evidence suggests that exposure to ambient air pollutants may serve as a critical environmental risk factor for the development of such infections. However, current findings are insufficient to define the magnitude or chronic implications of this association.

Recent epidemiological studies have begun to link specific pollutants to urogenital infections. For instance, a phenome-wide association analysis found that long-term exposure to PM_2.5_ and NO_2_ was associated with increased odds of cystitis [4], while short-term spikes in PM_2.5_ concentrations have been linked to elevated hospitalization rates for UTIs [5]. Parallel research in male reproductive health further indicates that chronic PM_2.5_ exposure may reduce sperm motility and concentration [6, 7], a finding also supported by recent meta-analyses [13]. Nevertheless, risk estimates varied widely between studies. In Canada, a time-series study found that an interquartile range increase in same-day NO_2_ (8.8 ppb) was associated with a 4.0% increase in emergency visits for urogenital diseases among women over 60 years of age (RR = 1.040, 95% CI: 1.028–1.052) [14]. In contrast, a two-stage study in Beijing observed a 7.5% increase in urinary hospitalization rates associated with similar increases in PM_2.5_ over a 0–10 day lag period [15], while a national time-series study across China reported less than a 1% increase per 10 µg/m^3^ rise in same-day PM_2.5_ [16]. Data from the US Medicare cohort further indicated that a 5 µg/m^3^ annual increase in PM_2.5_ corresponded to a 7.6% rise in first hospitalization risk for renal and urinary system diseases (HR = 1.076, 95% CI: 1.071–1.081) [17]. These inconsistencies arise from several sources, including variability in exposure windows (daily versus annual), differences in exposure assessment approaches (ecological versus individual modelling), heterogeneity in study populations, and inconsistent outcome definitions (case definitions ranging from laboratory-confirmed infections to hospitalisation records or ICD-coded symptoms). As a result, despite an overall positive trend, the true magnitude and certainty of the relationship between long-term pollutant exposure and urogenital infections remain elusive.

To address these critical Limitations and establish a clearer understanding of long-term environmental risk, there is an urgent need for large-scale, prospective cohort studies specifically designed to evaluate the association between air pollution and the incidence of UTIs and MRSIs. The present study aims to fill this gap by leveraging the UK Biobank, a well-characterized prospective cohort comprising nearly 500,000 participants with extensive long-term follow-up and detailed health and environmental exposure data [18]. By systematically assessing long-term exposure to key air pollutants (PM_2.5_, PM_coarse_, PM_10_, NO_2_, and NO_x_) in relation to clinically defined incident UTIs and MRSIs, our study seeks to overcome prior methodological limitations. Through robust exposure modeling and validated outcome ascertainment, this investigation intends to clarify the chronic effects of air pollution on urogenital infections and contribute applicable evidence to guide future public health policy and preventive strategies.

## Methods

### Sample and study design

The UK Biobank is a large-scale, community-based cohort comprising 500,000 UK residents, aged 37 to 73 years, who are registered with the National Health Service. Initial assessments were conducted across 22 centers in the UK between 2006 and 2010. This study has received approval from the Northwest Multicenter Research Ethics Committee (REC reference: 21/NW/0157) and adheres to the principles outlined in the Helsinki Declaration. Our research was carried out under Application Number 532,564. For additional details, please refer to the UK Biobank website: (http://www.ukbiobank.ac.uk/).

**Fig. 1:**
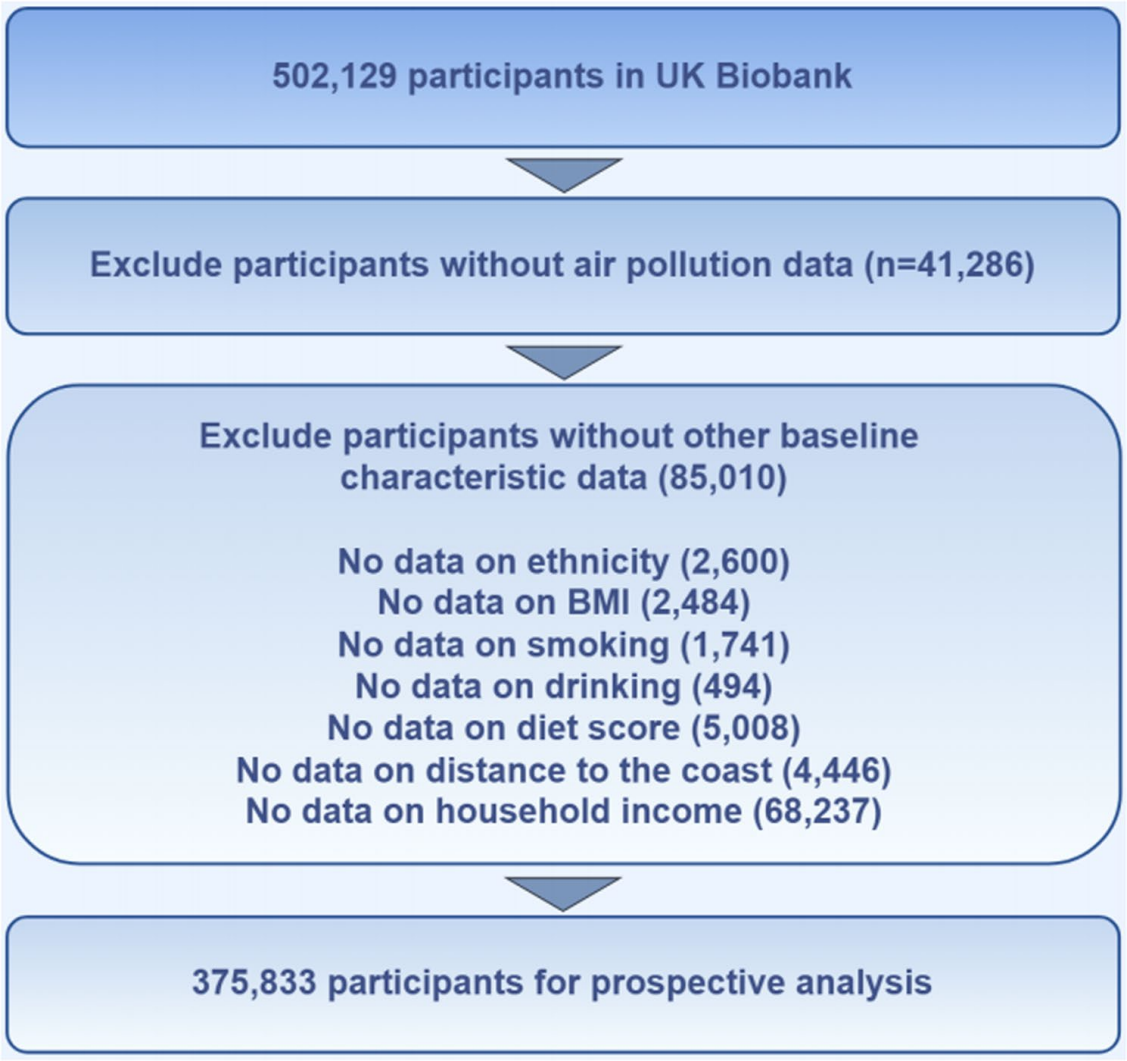
Overview of study design

Within the scope of this research, the baseline was established as the point when participants initially engaged with the evaluation center. Figure 1 presents the study’s structure, encompassing both inclusion and exclusion criteria for participants. In summary, following the exclusion of participants lacking complete air pollutant data (*N* = 41,286) and those with absent covariate details (*N* = 85,010), a total of 375,833 participants were incorporated into the analysis cohort.

### Air pollution assessment

The atmospheric contaminants assessed in this study encompassed inhalable PM_2.5_, coarse particulate matter (PM_coarse_) with aerodynamic diameters between 2.5 and 10 μm, PM_10_, nitrogen dioxide (NO_2_), and nitrogen oxides (NO_x_). The annual average concentrations of air pollutants for 2010 were estimated by employing land use regression models created by the European Cohort Study of Air Pollution Effects (ESCAPE) research team [19]. These models combined participants’ home addresses from the initial assessment with data from ESCAPE monitoring stations and Geographic Information System -generated predictor variables such as traffic density, population density, terrain, and land use to estimate variations in ambient air pollution levels across different locations [20]. Details regarding the creation and verification of these models are available in other sources (see http://www.escapeproject.eu/). Validation through cross-checking demonstrated strong model performance, with R² values of 77% for PM_2.5_, 57% for PM_coarse_, 88% for PM_10_, 87% for NO_2_, and 88% for NO_x_.

### Assessment of utis and MRSIs

UTIs cases were identified using validated hospitalization data and the International Classification of Diseases, Tenth Revision (ICD-10) coding system: N11.0 (non-obstructive reflux-related chronic pyelonephritis), N11.1 (chronic obstructive pyelonephritis), N13.6 (pyonephrosis), N15.1 (renal abscess and perirenal abscess), N30 (cystitis), N33.0 (tuberculous cystitis), N34.0 (urethral abscess), N34.1 (non-specific urethritis), N34.2 (other urethritis), and N39.0 (urinary tract infection, unspecified site). MRSIs were defined using ICD-10 codes N41 (inflammatory diseases of prostatitis) and N45 (orchitis and epididymitis). MRSIs analyses were Limited to male participants and that gender was retained as a covariate to maintain model consistency across outcomes. The follow-up time was calculated from the initial evaluation date until the earliest occurrence of death, diagnosis of UTIs or MRSIs, loss to follow-up, or the end of the study period, with the longest follow-up extending to 15 years.

### Assessment of covariates

Based on prior literature, potential confounding factors that could influence air pollution levels or the risk of UTIs and MRSIs were identified as covariates. These included population features (age, gender, ethnicity, and education), daily habits and health-related factors (smoking, drinking, physical activity, body mass index [BMI], and healthy diet score), as well as household attributes (household income and residence location distance to the coast) [21–23]. Age was treated as a continuous variable, determined based on the birth date and the initial evaluation date. At baseline, participant-reported gender was divided into women or men. Ethnicity was grouped into two categories: white and non-white. BMI (kg/m^2^) was classified into four categories according to WHO standards: Underweight (< 18.5), Normal (18.5–24.9), Overweight (25.0–29.9), and Obese (≥ 30.0) [24]. Education level was categorized as college, other, or unknown. Smoking and drinking behaviors were classified as never, previously, or currently. Regular physical activity was considered as participating in no less than 150 min of moderate-intensity exercise or 75 min of high-intensity exercise every week, or engaging in moderate-intensity physical activity for no fewer than 5 days each week, or undertaking vigorous activity once a week [25]. The Healthy Eating Score was composed of seven elements from the most recent American Dietary Guidelines, which are vegetables, fruits, fish, whole grains, refined grains, processed meats, and unprocessed meats. A comprehensive score was derived by aggregating the points allocated to each dietary component that satisfied the recommended intake target, with a maximum score of 7, where higher scores indicated healthier eating habits [26, 27]. Table S1 details the components and scoring system used in this calculation. Household income was divided into five categories: <£18,000, £18,000–£30,999, £31,000–£51,999, £52,000–£100,000, and >£100,000. The distance between all participant’s home and the coast was measured in kilometers (km).

### Statistical analysis

The baseline characteristics for continuous variables are presented as means with standard deviations (SD), while categorical variables are summarized as percentages. The UK Biobank data were not weighted, as the cohort design does not fully represent the broader community. We utilized Cox proportional hazards regression models to evaluate the link between long-term exposure to air pollutants and the risk of UTIs and MRSIs, adjusting for covariates in two distinct models. Model A included adjustments for age and gender, whereas Model B also accounted for ethnicity, education, BMI, household income, smoking status, drinking status, healthy diet score, physical activity, and residence location distance to the coast. We employed a restricted cubic spline (RCS) model using the ‘rms’ package to examine potential nonlinear relationships between air pollutant exposure and infection risks, with knots automatically placed at the 5th, 35th, 65th, and 95th percentiles of pollutant concentrations to optimally capture exposure-response patterns [28]. Additionally, in Line with prior studies, the effect estimates were presented for each pollutant as a 5 µg/m^3^ increase for PM_2.5_ and PM_coarse_, a 10 µg/m^3^ increase for PM_10_ and NO_2_, and a 20 µg/m^3^ increase for NO_x_ [29]. We also offered association estimates for each pollutant for each interquartile range (IQR) to enhance comparability across pollutants. Schoenfeld residuals method was used to test the proportional hazards assumptions for the Cox model and no apparent violations were found.

Stratified analysis was performed based on age, gender, ethnicity, BMI, education level, smoking status, physical activity, drinking status, diet score, household income, and distance from the coast to explore potential effect modifiers. The relationship between air pollution variables and stratification factors was evaluated by including the interaction terms in the Cox proportional hazards model. The *P*-value for the interaction was derived to ascertain if the impact of air pollution on the risk of UTIs and MRSIs differed across subgroups. A *P*-value less than 0.05 for the interaction term implies heterogeneity among subgroups, signifying that the effect of air pollution on the outcomes varies across these groups and often suggests the existence of an interaction effect.

Sensitivity analysis was performed to assess the robustness of our findings. First, to minimize the possibility of reverse causality, we reanalyzed the Link involving air pollutants and UTIs and MRSIs, excluding cases that emerged during the initial 3-year follow-up period. Second, to lessen possible misclassification of air pollution exposure resulting from residential relocation, we restricted the analysis to participants who had resided at the same address for a minimum of 5 years. Third, we carried out the analysis excluding outlier values of air pollutants, which were considered as values beyond the 1 st and 99th percentiles. Statistical analyses were performed with R software (version 4.3.2). P values less than 0.05 were considered statistically significant.

## Result

### Baseline characteristics of the participants

Over a median follow-up duration of 12.4 years, a total of 26,279 participants were identified as having UTIs and 3,545 with MRSIs among the 375,833 study participants. Table 1 shows the basic features of the study group at the start. The average (SD) age (with standard deviation in parentheses) of those with UTIs was 60.33 (7.20) years, with 14,567 (55.43%) being female. The majority of participants were white (25,054, 95.34%), and 12,420 (47.26%) had education at the “Other levels” category. The average (SD) distance from participants’ residences to the coast was 41.81 (27.86) km. A substantial proportion reported never smoking (12,601, 47.95%), current drinking (23,096, 87.89%), regular physical activity (14,547, 55.36%), being overweight (10,852, 41.30%), and an average (SD) healthy eating score of 2.80 (1.28).

**Table 1:** Baseline characteristics of participants in the UK biobank study

Characteristic	UTI		MRSI	
	Yes	No	Yes	No
	(*N* = 26,279)	(*N* = 349,554)	(*N* = 3,545)	(*N* = 372,288)
Age (years)				
Mean (SD)	60.33 (7.20)	56.29 (8.08)	59.30 (7.30)	56.55 (8.09)
Gender				
Female	14,567 (55.43%)	190,348 (54.45%)	0 (0%)	204,915 (55.04%)
Male	11,712 (44.57%)	159,206 (45.55%)	3,545 (100%)	167,373 (44.96%)
Ethnicity				
Non White	1,225 (4.66%)	18,515 (5.30%)	175 (4.94%)	19,565 (5.26%)
White	25,054 (95.34%)	331,039 (94.70%)	3,370 (95.06%)	352,723 (94.74%)
BMI				
Underweight	152 (0.58%)	1,818 (0.52%)	6 (0.17%)	1,964 (0.53%)
Normal	6,652 (25.31%)	115,920 (33.16%)	798 (21%)	121,774 (32.71%)
Overweight	10,852 (41.30%)	148,669 (42.53%)	1,760 (49.65%)	157,761 (42.38%)
Obese	8,623 (32.81%)	83,147 (23.79%)	981 (27.67%)	90,789 (24.39%)
Education				
Unknown	7,644 (29.09%)	60,734 (17.37%)	844 (23.81%)	67,534 (18.14%)
College	6,215 (23.65%)	113,109 (32.36%)	1,070 (30.18%)	118,254 (31.76%)
Other levels	12,420 (47.26%)	175,711 (50.27%)	1,631 (46.01%)	186,500 (50.10%)
Smoking status				
Never	12,601 (47.95%)	193,393 (55.33%)	1,633 (46.06%)	204,361 (54.89%)
Previous	10,620 (40.41%)	120,754 (34.55%)	1,458 (41.13%)	129,916 (34.90%)
Current	3,058 (11.64%)	35,407 (10.13%)	454 (12.81%)	38,011 (10.21%)
Physical activity				
Non	11,732 (44.64%)	135,928 (38.89%)	1,381 (38.96%)	146,279 (39.29%)
Yes	14,547 (55.36%)	213,626 (61.11%)	2,164 (61.04%)	226,009 (60.71%)
Drinking status				
Never	1,633 (6.21%)	14,347 (4.10%)	115 (3.24%)	15,865 (4.26%)
Previous	1,550 (5.90%)	11,627 (3.33%)	163 (4.60%)	13,014 (3.50%)
Current	23,096 (87.89%)	323,580 (92.57%)	3,267 (92.16%)	343,409 (92.24%)
Diet score				
Mean (SD)	2.80 (1.28)	2.80 (1.29)	2.51 (1.34)	2.81 (1.29)
Household income (£)				
Less than 18,000	6,138 (23.36%)	79,864 (22.85%)	854 (24.09%)	85,148 (22.87%)
18,000 to 30,999	6,695 (25.48%)	89,042 (25.47%)	858 (24.20%)	94,879 (25.49%)
31,000 to 51,999	6,874 (26.16%)	90,909 (26.01%)	924 (26.06%)	96,859 (26.02%)
52,000 to 100,000	5,224 (19.88%)	70,920 (20.29%)	711 (20.06%)	75,433 (20.26%)
Greater than 100,000	1,348 (5.13%)	18,819 (5.38%)	198 (5.59%)	19,969 (5.36%)
Residence location distance to the coast (km)				
Mean (SD)	41.81 (27.86)	41.31 (27.82)	41.50 (27.75)	41.34 (27.82)

*UTI* urinary tract infection, *MRSI* male reproductive system infection, *Q1* first Quartile, *Q3* third Quartile, *BMI* body mass index, *SD* standard deviations

For participants with MRSIs, the average (SD) age was 59.30 (7.30) years, with 3,370 (95.06%) being white, and 1,631 (46.01%) having education at the “Other levels” category. The average (SD) distance from residence to the coast was 41.50 (27.75) km. A large proportion of these participants reported never smoking (1,633, 46.06%), current drinking (3,267, 92.16%), regular physical activity (2,164, 61.04%), being overweight (1,760, 49.65%), and an average (SD) healthy eating score of 2.51 (1.34). Table S2 illustrates the Spearman’s correlation coefficients for a range of air pollutants. Variance inflation factor (VIF) analysis indicated that all VIF values for explanatory variables in the model were between 1 and 2, suggesting no substantial multicollinearity among the covariates. Consequently, all variables were kept in the final model, further bolstering the robustness and reliability of our results.

### Air pollutants and utis

**Fig. 2:**
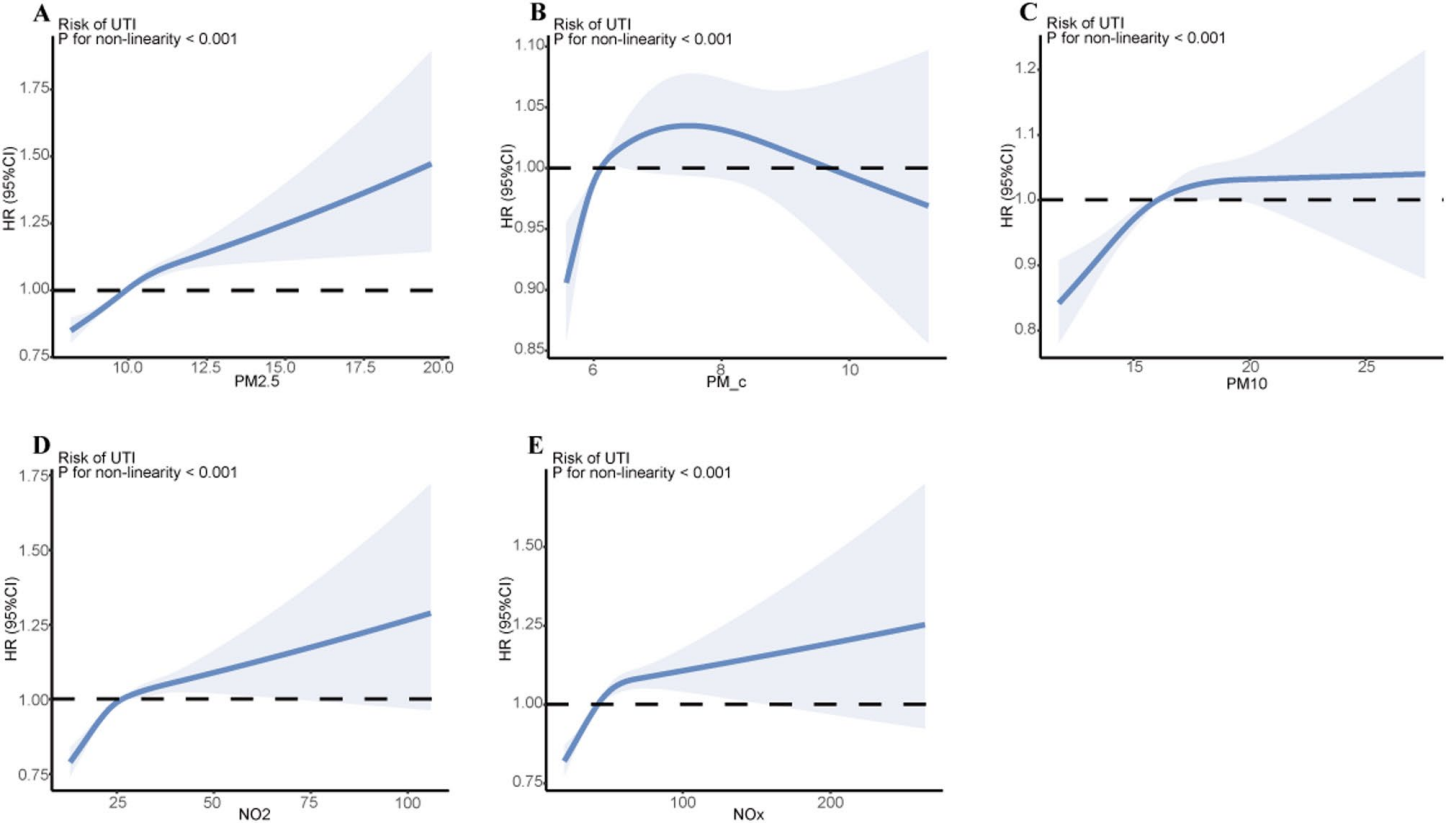
Exposure − response relationship between air pollutants and the risk of UTIs. (**a**) PM_2.5_ and UTIs; (**b**) PM_coarse_ and UTIs; (**c**) PM_10_ and UTIs; (**d**) NO_2_ and UTIs; and (**e**) NO_x_ and UTIs. HRs adjusted for age (as the underlying time scale), sex, ethnicity, BMI, education, household income, alcohol drinking status, smoking status, healthy diet score, physical activity, and residence location distance to the coast. Abbreviations: NO_2_, nitrogen dioxide; NO_x_, nitrogen oxide; PM_2.5_, particulate matter with aerodynamic diameters of ≤ 2.5 μm; PM_coarse_, particulate matter with aerodynamic diameters between 2.5 and 10 μm; PM_10_, particulate matter with aerodynamic diameters of ≤ 10 μm

In models with multiple adjustments, we found that greater exposure to air pollutants was associated with a higher risk of UTIs, as shown in Table 2. More precisely, for every 5 µg/m^3^ increase in PM_2.5_ and PM_coarse_, and every 10 µg/m^3^ increase in PM_10_, the adjusted risks of UTIs were elevated by 43% (95% CI: 34–53%, *P* < 0.001), 17% (95% CI: 8–27%, *P* < 0.001), and 24% (95% CI: 15–34%, *P* < 0.001), respectively. Additionally, each 10 µg/m^3^ increase in NO_2_ and 20 µg/m^3^ increase in NO_x_ corresponded to a 10% (95% CI: 8–12%, *P* < 0.001) and 9% (95% CI: 7–11%, *P* < 0.001) higher risk of UTIs, respectively. Compared to participants in the group with the least air pollution exposure, those exposed to greater degrees of PM_2.5_, PM_coarse_, PM_10_, NO_2_, and NO_x_ had significantly increased risks of UTIs (Table 2). For UTIs, the RCS curves showed that the HRs for PM_2.5_, PM_coarse_, PM_10_, NO_2_, and NO_x_ increased with higher pollutant concentrations, particularly evident at higher exposure levels (Fig. 2). For example, the HR for PM_2.5_ showed a steeper upward trend when concentrations exceeded 15 µg/m^3^, indicating a more pronounced risk increase at higher exposure.

**Table 2:** Associations between long-term exposure to ambient air pollution and the risk of urinary tract infection

Pollution	Increase	Events	Total/Person-years	Model a HR (95% CI)	*P*	Model b HR (95% CI)	*P*
PM _2.5_	per 5 µg/m^3^ increment	18,477	352,469/13,255	1.71 (1.60, 1.83)	< 0.001	1.43 (1.34, 1.53)	< 0.001
	Q1(8.17,9.29)	4279	90,078/3384	ref	ref	ref	ref
	Q2(9.29,9.93)	4480	88,212/3306	1.09 (1.05, 1.14)	< 0.001	1.05 (1.01, 1.09)	0.026
	Q3(9.93,10.6)	4793	88,573/3325	1.20 (1.15, 1.25)	< 0.001	1.11 (1.07, 1.16)	< 0.001
	Q4(10.6,21.3)	4925	85,606/3239	1.34 (1.28, 1.39)	< 0.001	1.21 (1.16, 1.26)	< 0.001
	*P* for trend	-	-	-	< 0.001	-	< 0.001
PM _coarse_	per 5 µg/m^3^ increment	18,477	352,469/13,255	1.25 (1.15, 1.35)	< 0.001	1.17 (1.08, 1.27)	< 0.001
	Q1(5.57,5.84)	4440	90,132/3408	ref	ref	ref	ref
	Q2(5.84,6.11)	4561	88,673/3336	1.07 (1.02, 1.11)	0.002	1.04 (1.00, 1.09)	0.046
	Q3(6.11,6.64)	4667	86,936/3245	1.14 (1.10, 1.19)	< 0.001	1.10 (1.06, 1.15)	< 0.001
	Q4(6.64,12.8)	4809	86,728/3266	1.17 (1.13, 1.22)	< 0.001	1.13 (1.08, 1.17)	< 0.001
	*P* for trend	-	-	-	< 0.001	-	< 0.001
PM _10_	per 10 µg/m^3^ increment	18,477	352,469/13,255	1.39 (1.29, 1.49)	< 0.001	1.24 (1.15, 1.34)	< 0.001
	Q1(11.8,15.2)	4368	89,198/3361	ref	ref	ref	ref
	Q2(15.2,16)	4736	88,870/3361	1.10 (1.06, 1.15)	< 0.001	1.06 (1.02, 1.11)	0.004
	Q3(16,17)	4593	87,313/3259	1.13 (1.09, 1.18)	< 0.001	1.08 (1.03, 1.12)	< 0.001
	Q4(17,31.4)	4780	87,088/3273	1.19 (1.14, 1.24)	< 0.001	1.12 (1.08, 1.17)	< 0.001
	*P* for trend	-	-	-	< 0.001	-	< 0.001
NO _2_	per 10 µg/m^3^ increment	20,063	378,325/14,301	1.15 (1.13, 1.17)	< 0.001	1.10 (1.08, 1.12)	< 0.001
	Q1(12.9,21.5)	4522	96,327/3640	ref	ref	ref	ref
	Q2(21.5,26.2)	5143	94,935/3597	1.17 (1.13, 1.22)	< 0.001	1.11 (1.07, 1.15)	< 0.001
	Q3(26.2,31.3)	5289	94,329/3575	1.25 (1.20, 1.30)	< 0.001	1.16 (1.11, 1.21)	< 0.001
	Q4(31.3,108)	5109	92,734/3489	1.33 (1.28, 1.39)	< 0.001	1.22 (1.17, 1.27)	< 0.001
	*P* for trend	-	-	-	< 0.001	-	< 0.001
NO _x_	per 20 µg/m^3^ increment	20,063	378,325/14,301	1.13 (1.12, 1.15)	< 0.001	1.09 (1.07, 1.11)	< 0.001
	Q1(19.7,34.4)	4515	96,315/3637	ref	ref	ref	ref
	Q2(34.4,42.4)	5042	94,736/3581	1.16 (1.11, 1.20)	< 0.001	1.10 (1.06, 1.14)	< 0.001
	Q3(42.4,50.8)	5170	94,329/3563	1.24 (1.19, 1.29)	< 0.001	1.14 (1.10, 1.19)	< 0.001
	Q4(50.8,266)	5336	92,945/3521	1.37 (1.32, 1.43)	< 0.001	1.24 (1.19, 1.29)	< 0.001
	*P* for trend	-	-	-	< 0.001	-	< 0.001

Model a: age (as the underlying time scale) and gender

Model b: age (as the underlying time scale), sex, ethnicity, BMI, education, household income, smoking status, drinking status, diet score, physical activity, and residence location distance to the coast

*Abbreviations*: *CI* confidence interval, *HR* hazard ratio, *NO*_*2*_ nitrogen dioxide, *NO*_*x*_ nitrogen oxide, *PM*_*2.5*_ particulate matter with aerodynamic diameters of ≤2.5 μm, *PM*_*coarse*_ particulate matter with aerodynamic diameters between 2.5 and 10 μm, *PM*_*10*_ particulate matter with aerodynamic diameters of ≤10 μm, *Q* quartile

Stratified analysis revealed significant effect modification for PM_coarse_ and UTIs in older adults (age > 60 years, adjusted HR: 1.21, 95% CI: 1.13–1.30, *P* < 0.001) and females (adjusted HR: 1.19, 95% CI: 1.11–1.28, *P* < 0.001), whereas associations were weaker in non-white and underweight subgroups (all *P* > 0.05; Fig S1). Other pollutants showed consistent positive trends across most subgroups, with no significant interactions (all P for interaction > 0.05), as detailed in Supplementary Figs S2–S5.

### Air pollutants and MRSIs

**Fig. 3:**
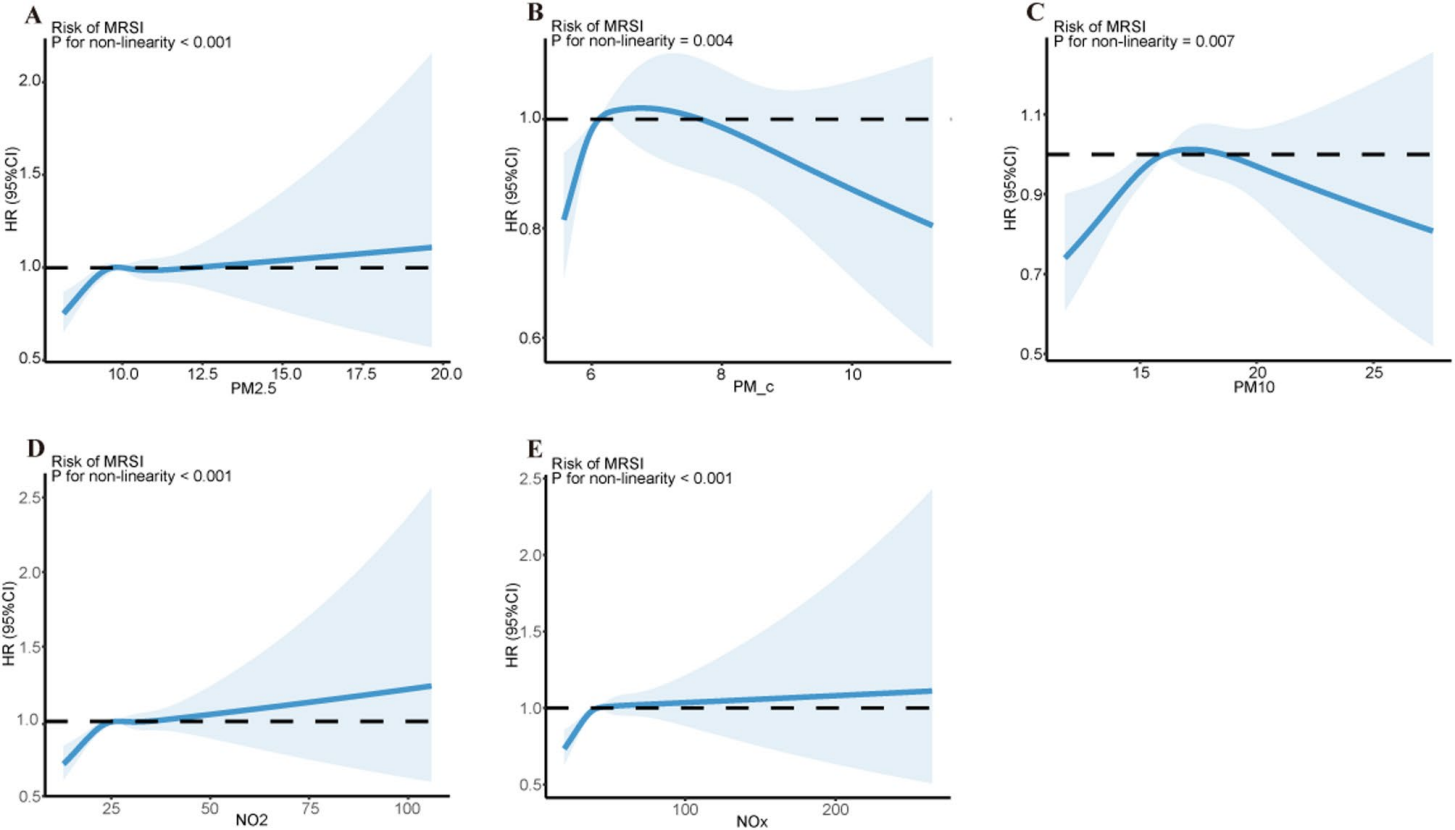
Exposure − response relationship between air pollutants and the risk of MRSIs. (**a**) PM_2.5_ and MRSIs; (**b**) PM_coarse_ and MRSIs; (**c**) PM_10_ and MRSIs; (**d**) NO_2_ and MRSIs; and (**e**) NO_x_ and MRSIs. HRs adjusted for age (as the underlying time scale), sex, ethnicity, BMI, education, household income, alcohol drinking status, smoking status, healthy diet score, physical activity, and residence location distance to the coast. Abbreviations: NO_2_, nitrogen dioxide; NO_x_, nitrogen oxide; PM_2.5_, particulate matter with aerodynamic diameters of ≤ 2.5 μm; PM_coarse_, particulate matter with aerodynamic diameters between 2.5 and 10 μm; PM_10_, particulate matter with aerodynamic diameters of ≤ 10 μm

In a similar vein, our findings indicated that individuals with greater exposure to air pollutants had an elevated risk of MRSIs when compared to those in the lowest exposure quartile. Specifically, each 5 µg/m^3^ increase in PM_2.5_ was associated with a 30% higher risk of MRSIs (adjusted HR: 1.30, 95% CI: 1.09–1.57, *P* = 0.004), while a 10 µg/m^3^ increase in PM_10_ was Linked to a 24% elevated risk (adjusted HR: 1.24, 95% CI: 1.01–1.52, *P* = 0.040). Exposure to NO_2_ and NO_x_ also showed significant associations, with each 10 µg/m^3^ increase in NO_2_ corresponding to an 12% higher risk (adjusted HR: 1.12, 95% CI: 1.06–1.17, *P* < 0.001) and each 20 µg/m^3^ increase in NO_x_ associated with an 8% higher risk (adjusted HR: 1.08, 95% CI: 1.03–1.13, *P* < 0.001) (Table 3). For MRSIs, the curves demonstrated that PM_2.5_, NO_2_, and NO_x_ exposures were associated with non-linear increases in risk, with PM_2.5_ showing a notable rise in HR when concentrations exceeded 12.5 µg/m^3^ (Fig. 3).

**Table 3:** Associations between long-term exposure to ambient air pollution and the risk of male reproductive tract infection

Pollution	Increase	Events	Total/Person-years	Model a HR (95% CI)	*P*	Model b HR (95% CI)	*P*
PM _2.5_	per 5 µg/m^3^ increment	2510	372,288/13,572	1.40 (1.17, 1.68)	< 0.001	1.30 (1.09, 1.57)	0.004
	Q1(8.17,9.29)	578	94,613/3455	ref	ref	ref	ref
	Q2(9.29,9.93)	646	92,970/3382	1.16 (1.04, 1.30)	< 0.001	1.14 (1.02, 1.28)	0.019
	Q3(9.93,10.6)	661	93,749/3409	1.21 (1.08, 1.35)	< 0.001	1.18 (1.05, 1.32)	0.005
	Q4(10.6,21.3)	625	90,956/3325	1.21 (1.08, 1.36)	< 0.001	1.16 (1.04, 1.30)	0.010
	*P* for trend	-	-	-	< 0.001	-	0.010
PM _coarse_	per 5 µg/m^3^ increment	2510	372,288/13,572	1.14 (0.92, 1.4)	0.219	1.11 (0.90, 1.37)	0.331
	Q1(5.57,5.84)	575	94,999/3488	ref	ref	ref	ref
	Q2(5.84,6.11)	640	93,536/3415	1.14 (1.02, 1.28)	0.023	1.13 (1.01, 1.26)	0.034
	Q3(6.11,6.64)	648	91,888/3322	1.21 (1.08, 1.35)	< 0.001	1.19 (1.06, 1.33)	0.003
	Q4(6.64,12.8)	647	91,865/3347	1.19 (1.06, 1.33)	< 0.001	1.16 (1.04, 1.30)	0.008
	*P* for trend	-	-	-	0.014	-	0.037
PM _10_	per 10 µg/m^3^ increment	2510	372,288/13,572	1.30 (1.06, 1.59)	0.011	1.24 (1.01, 1.52)	0.040
	Q1(11.8,15.2)	592	93,893/3437	ref	ref	ref	ref
	Q2(15.2,16)	628	93,981/3443	1.08 (0.97, 1.21)	0.181	1.07 (0.95, 1.19)	0.259
	Q3(16,17)	648	92,231/3339	1.17 (1.05, 1.31)	< 0.001	1.15 (1.02, 1.28)	0.017
	Q4(17,31.4)	642	92,183/3352	1.16 (1.03, 1.29)	0.010	1.13 (1.01, 1.26)	0.036
	*P* for trend	-	-	-	0.007	-	0.028
NO _2_	per 10 µg/m^3^ increment	2643	399,897/14,648	1.14 (1.09, 1.20)	< 0.001	1.12 (1.06, 1.17)	< 0.001
	Q1(12.9,21.5)	573	101,232/3720	ref	ref	ref	ref
	Q2(21.5,26.2)	688	100,435/3685	1.23 (1.10, 1.37)	< 0.001	1.21 (1.08, 1.35)	< 0.001
	Q3(26.2,31.3)	679	100,042/3667	1.25 (1.12, 1.40)	< 0.001	1.22 (1.09, 1.37)	< 0.001
	Q4(31.3,108)	703	98,188/3576	1.38 (1.24, 1.54)	< 0.001	1.33 (1.18, 1.48)	< 0.001
	*P* for trend	-	-	-	< 0.001	-	< 0.001
NO _x_	per 20 µg/m^3^ increment	2643	399,897/14,648	1.10 (1.05, 1.15)	< 0.001	1.08 (1.03, 1.13)	< 0.001
	Q1(19.7,34.4)	598	101,150/3715	ref	ref	ref	ref
	Q2(34.4,42.4)	665	100,197/3669	1.14 (1.02, 1.28)	0.018	1.12 (1.00, 1.25)	0.042
	Q3(42.4,50.8)	701	99,860/3651	1.25 (1.12, 1.39)	< 0.001	1.21 (1.09, 1.35)	< 0.001
	Q4(50.8,266)	679	98,690/3613	1.26 (1.13, 1.41)	< 0.001	1.21 (1.08, 1.35)	< 0.001
	*P* for trend	-	-	-	< 0.001	-	< 0.001

Model a: age (as the underlying time scale) and gender

Model b: age (as the underlying time scale), sex, ethnicity, BMI, education, household income, smoking status, drinking status, diet score, physical activity, and residence location distance to the coast

*Abbreviations*: *CI* confidence interval, *HR* hazard ratio, *NO*_*2*_ nitrogen dioxide, *NO*_*x*_ nitrogen oxide, *PM*_*2.5*_ particulate matter with aerodynamic diameters of ≤ 2.5 μm, *PM*_*coarse*_ particulate matter with aerodynamic diameters between 2.5 and 10 μm, *PM*_*10*_ particulate matter with aerodynamic diameters of ≤ 10 μm, *Q* quartile

In stratified analyses, significant positive associations between air pollutants and MRSI risk were observed in specific subgroups: PM_2.5_ in adults > 60 years, those with overweight BMI, unknown education, current smokers, non-physically active, current drinkers, diet score < 3, household income <£18k or £52k–£100k, and residence > 43 km from coast (Fig S6); PM_coarse_ in those with household income >£100k and residence > 43 km from coast (Fig S7); PM_10_ in those with overweight BMI, previous drinkers, household income <£18k or >£100k, and residence > 43 km from coast (Fig S8); and NO_2_/NO_x_ in adults > 60 years, white individuals, those with overweight BMI, unknown education, never/current drinkers, diet score < 3, household income <£18k, and residence > 43 km from coast (Figs S9–S10). Significant interaction effects were limited to coastline distance for PM_coarse_, drinking status, household income, and coastline distance for PM_10_, and drinking status for NO_2_, with no significant interactions in other subgroups (interaction *P* > 0.05).

### Sensitivity analysis

Sensitivity analysis strongly supports the robustness of the observed association between air pollutants and both UTIs and MRSIs in the preliminary analysis. Specifically, excluding cases that occurred during the initial 3-year follow-up period revealed no significant change in the relationship between air pollutants and the risk of UTIs and MRSIs (Tables S3 and S4). When participants who had resided in their current residences for fewer than 5 years were excluded, a positive correlation remained between air pollutants and the risk of UTIs and MRSIs (Tables S5 and S6). Similarly, excluding participants subjected to extreme concentrations of air pollutants did not change the association between air pollution and UTIs and MRSIs (Tables S7 and S8).

## Discussion

Previous studies have established a link between air pollution and various urinary system disorders, including urolithiasis [30], urinary incontinence [31], prostate cancer [32], bladder cancer and renal cancer [33]. Nevertheless, only a limited number of studies have specifically explored the long-term impacts of air pollution on UTIs. One study assessed the hospitalization risk and associated costs of short-term exposure to PM_2.5_ in 214 mutually exclusive disease groups, finding a connection between UTIs and short-term exposure to PM_2.5_ [5]. Additionally, cystitis, a key component of UTIs, has been found to be associated with exposure to both PM_2.5_ and NO_2_ in other studies [4]. Emerging evidence suggests potential mechanisms by which air pollution may influence UTIs. For instance, PM_coarse_ exposure has been linked to airway inflammation in children, raising the possibility of systemic inflammatory effects that could compromise urinary tract mucosal barrier function [34]. Heavy metals such as lead and cadmium, present in PM_10_ and PM_coarse_, may indirectly increase infection risk by inducing nephrotoxicity and damaging urinary tract tissues [35]. Similarly, NO_x_ can enter the bloodstream via the respiratory system, triggering systemic oxidative stress that may predispose individuals to UTIs [36].

Notably, however, no epidemiological research has specifically evaluated the association between air pollution and MRSIs, defined clinically in our study as prostatitis (ICD-10 N41) and orchitis/epididymitis (ICD-10 N45). Although prior research linking air pollution to male reproductive health exists, it predominantly focuses on non-infectious outcomes such as semen quality impairment and benign prostatic hyperplasia (BPH) [37–39]. These conditions, although relevant to male reproductive health, are not categorized as infections and therefore lie beyond the scope of our defined outcomes for MRSIs. Nevertheless, experimental data from animal models have indicated that particulate matter exposure can trigger testicular inflammation, suggesting a biologically plausible pathway that may underlie such associations in humans [40]. Among the existing studies on UTIs and male reproductive health, the majority employed cross-sectional designs with relatively limited sample sizes, though a few longitudinal studies have been reported. The variability in study design, outcome definitions, and exposure duration across these studies reduces their comparability and limits the strength of causal inference. In this context, our study provides new epidemiological evidence directly linking long-term exposure to ambient air pollution with clinically diagnosed UTIs and MRSIs.

Nonetheless, these studies have certain limitations that may contribute to inconsistent findings. Initially, the representativeness and accuracy of aligning disease and environmental information in cross-sectional studies require additional confirmation. Moreover, a critical gap exists in differentiating short-term vs. long-term exposure effects: most UTI-related studies analyzed short-term exposure, such as PM2.5 fluctuations and hospitalization risks [41], whereas only 12% of studies addressed long-term exposure [39]. This distinction is biologically significant, as short-term exposure likely triggers acute inflammatory responses, while long-term exposure may induce chronic oxidative stress, immune dysfunction, and vascular damage—mechanisms supported by our observation of non-linear dose-response relationships [42, 43].

Our results demonstrate consistent associations between long-term exposure to multiple ambient air pollutants and increased risks of both UTIs and MRSIs, including evidence of nonlinear exposure-response patterns. Importantly, except for PM_coarse_, the other pollutants assessed (PM_2.5_, PM_10_, NO_2_, and NO_x_) showed significant associations with MRSIs. These findings bridge an important knowledge gap, extending prior evidence on short-term NOx-cystitis associations to long-term impacts on reproductive health. We also identified complex, nonlinear exposure-response relationships between these pollutants and the risks of both UTIs and MRSIs. Considering that factors such as age, gender, BMI, and Lifestyle might confound the relationship between air pollution and the incidence of UTIs and MRSIs. To check the stability of our results, we did a subgroup analysis. While the Link involving specific air pollutants and UTIs and MRSIs differed among various subgroups, the general trend of the estimated impacts stayed the same, hinting at a steady positive link between these pollutants and the risks of UTIs and MRSIs. It should be pointed out that the lack of significance in some subgroups might be attributed to sample size constraints, as the interaction *P*-values for nearly all stratification factors, with a few exceptions, exceeded 0.05.

Although this study confirms the association between air pollution and the incidence of UTIs and MRSIs, the underlying mechanisms remain unclear, potentially involving vascular damage and oxidative stress (OS). Animal studies have demonstrated that exposure to PM_2.5_ triggers oxidative stress by activating the Nrf2/NF-κB pathway, leading to increased levels of inflammatory cytokines [42]. A study on female solid fuel users found a 32% higher white blood cell count in the blood compared to clean fuel users, with greater production of reactive oxygen species by neutrophils, lymphocytes, eosinophils, and alveolar macrophages [44, 45]. OS, in turn, can exacerbate systemic inflammation by promoting the production of pro-inflammatory cytokines [46]. In prostate tissue, the imbalance between OS and inflammation can lead to the accumulation of growth factors and inflammatory cytokines [47]. Systemic inflammation and the generation of oxygen free radicals may cause oxidative damage to the cell membranes of the urethra and bladder, contributing to UTIs and MRSIs. When the particle size of air pollutants is ≤ 10 μm, PM inhaled through the nasal cavity and lungs can reach the alveoli, and even smaller particles can penetrate deeper into the respiratory system. Particles smaller than 1 μm can enter the circulatory system, much like gas molecules, and reach the urinary system through the bloodstream. These particles may directly or indirectly affect renal function and alter the spectrum of urinary metabolites, potentially leading to vascular damage and inflammation in the urinary tract [48, 49].

Our study demonstrated a higher prevalence of UTIs among elderly individuals and women compared to younger populations and males. This epidemiological pattern can be attributed to several anatomical and physiological factors. Females exhibit a significantly shorter urethral length (approximately 4 cm) compared to males (20 cm), resulting in a reduced physical barrier against ascending bacterial colonization [9]. Furthermore, the anatomical proximity of the female urethral meatus to the anorectal region increases exposure to enteric microbiota, particularly Escherichia coli, which accounts for 80–90% of uncomplicated UTI cases [8]. In contrast, the male urinary tract possesses inherent protective mechanisms, including a longer urethral course and prostate gland secretions containing antimicrobial components such as zinc and cytokines [11]. These factors collectively contribute to a lower susceptibility to UTIs in male populations. The age-related predisposition to UTIs can be explained by multiple pathophysiological changes. Advancing age leads to structural alterations in the urinary tract, including bladder detrusor muscle atrophy, incomplete voiding, and urinary stasis, all of which facilitate bacterial proliferation [50]. Postmenopausal women face additional risk factors due to estrogen deficiency, which compromises the integrity of urothelial cells and reduces vaginal colonization by protective Lactobacillus species [51]. These physiological changes collectively contribute to the observed epidemiological patterns in UTI susceptibility.

Our study has several notable strengths. First, we employed a prospective cohort design to examine the impact of long-term air pollution exposure and its link to the risk of UTIs and MRSIs. Second, the large sample size from the UKB dataset significantly boosted our ability to detect effects, enabling a more precise assessment of the potential impacts of air pollution on the incidence of UTIs and MRSIs. Furthermore, the robustness of our findings persisted even after accounting for various confounding factors, strengthening the evidence for the relationship between air pollution and UTIs and MRSIs. Considering public health, our findings highlight the pressing need for stricter air quality rules and specific measures to lower exposure to air pollutants, which might ease the pressure on medical services due to UTIs and MRSIs. These results also emphasize the importance of integrating urological and reproductive health factors into environmental policies, particularly in highly urbanized or industrialized areas, and adopting a collaborative strategy involving multiple sectors to reduce the negative impacts caused by air pollution. To our knowledge, this is the largest longitudinal study to date to comprehensively assess the association between long-term exposure to multiple ambient air pollutants—including PM_2.5_, PM_coarse_, PM_10_, NO_2_, and NO_x_—and the incidence of UTIs and MRSIs in a general population. By using clinically validated outcomes and individual-level exposure assessment, this work offers critical new insights into the potential infectious sequelae of chronic air pollution exposure.

Nevertheless, this study has several Limitations. Firstly, air pollution is a dynamic and intricate blend of various human-made and natural pollutants, and the interactions among these pollutants make it challenging to evaluate the individual relationships of each component with UTIs and MRSIs. Secondly, although we adjusted for several confounding factors, the potential for residual confounding stemming from unmeasured or unknown variables is still present, and thus, the associations between air pollutants and UTIs and MRSIs remain subject to some uncertainty. Third, air pollution exposure was only measured at baseline, and we did not account for changes in air pollution exposure over time, which may influence the incidence of UTIs and MRSIs before and after cohort entry. Notably, using single-year pollution data may underrepresent long-term exposure dynamics, particularly for participants enrolled before 2010. Future studies with longitudinal pollution monitoring (e.g., annual updates) are required to verify our results. Additionally, while this study focused on five specific air pollutants—PM_2.5_, PM_coarse_, PM_10_, NO_2_, and NO_x,_ other pollutants such as ozone, carbon monoxide, and sulfur dioxide were excluded from the analysis. Therefore, the possible role of these unmeasured pollutants in the development of UTIs and MRSIs is still not clear. Future research should incorporate a broader range of air pollutants to investigate their effects on these conditions. Lastly, the assessment of air pollution levels was based exclusively on participants’ home addresses, without taking into account the time they spent in transportation, indoor settings, or workplaces. This approach might result in inaccurate exposure classification and could obscure the true relationship between air pollution and UTIs and MRSIs.

## Conclusion

In conclusion, our study has established a clear association between long-term exposure to air pollution and an increased risk of UTIs and MRSIs. These findings underscore the potential public health impact of environmental air pollution on the development of these conditions, highlighting the urgent need for further research in this area. Moreover, targeted environmental interventions aimed at reducing air pollution exposure could be crucial in mitigating the burden of UTIs and MRSIs.

## Supplementary Information

Supplementary Material 1.

## Data Availability

The UK Biobank data are available on application to the UK Biobank.
