# Supplementary material for: The impact of prolonged air pollution exposure on urinary tract and male reproductive system infections: insights from a large-scale prospective cohort study
Source: BMC Public Health. 2025 Oct 8;25:3401. doi: 10.1186/s12889-025-24660-6 (PMC12505862; doi:10.1186/s12889-025-24660-6)
Supplement: Supplementary file 1 — Supplementary Material 1. [file 12889_2025_24660_MOESM1_ESM.docx]

**Table S1** Components of diet score in the UK Biobank.

| Diet component | Intake goal | Field ID in UK Biobank | Amount per serving | |
| --- | --- | --- | --- | --- |
| Fruits | ≥3 servings/day | 1309 (pieces fresh fruit/day) 1319 (pieces dried fruit/day) | 1309 —1 piece  1319 —5 pieces | |
| Vegetable | ≥3 servings/day | 1289 (tablespoons cooked vegetables/day) 1299 (salad/raw vegetables/day) | 3 heaped tablespoons | |
| Fish | ≥2 servings/week | 1329 (oily fish/week)  1339 (non-oily fish/week) | Once/week | |
| Whole grains | ≥3 servings/day | 1438, 1448 (wholemeal or wholegrain bread slices/week) 1458, 1468 (bran/oat/muesli cereal bowls/week) | 1438/1448 —1  1458/1468 —1 | slice/day bowl/day |
| Refined grains | ≤2 servings/day | 1438, 1448 (white, brown, other bread slices/week) 1458, 1468 (biscuit, other cereals/week) | 1438/1448 —1  1458/1468 —1 | slice/day bowl/day |
| Processed meats | ≤1 servings/week | 1349 (processed meat/week or daily) 3680 (age when last ate meat) | 1349 —1 piece/day  3680 —0 pieces/day if indicated having never eaten meat | |
|  |  | 1359 (poultry/week or day) |  | |
|  |  | 1369 (beef/week or day) | 1359–1389 —once/week | |
| Unprocessed meats | ≤2 servings/week | 1379 (lamb or mutton/week or day) 1389 (pork/week or day) | 3680 —0 pieces/day if indicated having never eaten meat | |
|  |  | 3680 (age when last ate meat) |  | |

**Table S2** Spearman correlation coefficients among exposures related to the air pollutants.

| Variables | PM_2.5_ | PM_coarse_ | PM_10_ | NO_2_ | NO_x_ |
| --- | --- | --- | --- | --- | --- |
| PM_2.5_ | 1.00 | - | - | - | - |
| PM_coarse_ | 0.28 | 1.00 | - | - | - |
| PM_10_ | 0.57 | 0.78 | 1.00 | - | - |
| NO_2_ | 0.85 | 0.29 | 0.54 | 1.00 | - |
| NO_x_ | 0.87 | 0.29 | 0.55 | 0.92 | 1.00 |

PM_2.5_, particulate matter with aerodynamic diameter ≤2.5 mm; PM_coarse_, particulate matter with an aerodynamic diameter between 2.5 and 10 mm; PM_10_, particulate matter with an aerodynamic diameter ≤10 mm; NO_2_, nitrogen dioxide; NO_x_, nitrogen oxides.

**Table S3** Multivariable‑adjusted hazard ratios (95% CI) for air pollution concentrations with the risk of UTI in the UK Biobank study, after excluding cases of UTI that occurred within 3 years of follow-up.

| Pollution | Increase | Events | Total/Person-years | Model a  HR (95% CI) | P | Model b  HR (95% CI) | P |
| --- | --- | --- | --- | --- | --- | --- | --- |
| PM_2.5_ | per 5 μg/m^3^ increment | 17378 | 346674/13155 | 1.71 (1.60, 1.83) | < 0.001 | 1.44340421804551 (1.35, 1.55) | < 0.001 |
|  | Q1 (8.17,9.29) | 4038 | 88712/3360 | ref | ref | ref | ref |
|  | Q2 (9.29,9.93) | 4199 | 86856/3283 | 1.09 (1.04, 1.14) | < 0.001 | 1.04621805313084 (1.00, 1.09) | 0.041 |
|  | Q3 (9.93,10.6) | 4503 | 87026/3298 | 1.20 (1.15, 1.25) | < 0.001 | 1.11846547106372 (1.07, 1.17) | < 0.001 |
|  | Q4 (10.6,21.3) | 4638 | 84080/3214 | 1.34 (1.28, 1.40) | < 0.001 | 1.21611563044718 (1.17, 1.27) | < 0.001 |
|  | P for trend | - | - | - | < 0.001 | - | < 0.001 |
| PM_coarse_ | per 5 μg/m^3^ increment | 17378 | 346674/13155 | 1.24 (1.15, 1.35) | < 0.001 | 1.17657807706179 (1.09, 1.27) | < 0.001 |
|  | Q1 (5.57,5.84) | 4194 | 88699/3383 | ref | ref | ref | ref |
|  | Q2 (5.84,6.11) | 4313 | 87206/3311 | 1.077 (1.02, 1.12) | 0.002 | 1.04742349176741 (1.00, 1.09) | 0.033 |
|  | Q3 (6.11,6.64) | 4358 | 85484/3220 | 1.13 (1.09, 1.18) | < 0.001 | 1.09756500224039 (1.05, 1.15) | < 0.001 |
|  | Q4 (6.64,12.8) | 4513 | 85285/3241 | 1.17 (1.12, 1.22) | < 0.001 | 1.12465926670002 (1.08, 1.17) | < 0.001 |
|  | P for trend | - | - | - | < 0.001 | - | < 0.001 |
| PM_10_ | per 10 μg/m^3^ increment | 17378 | 346674/13155 | 1.39 (1.29, 1.50) | < 0.001 | 1.25270662044343 (1.16, 1.35) | < 0.001 |
|  | Q1 (11.8,15.2) | 4130 | 87781/3336 | ref | ref | ref | ref |
|  | Q2 (15.2,16) | 4446 | 87423/3336 | 1.09 (1.05, 1.14) | < 0.001 | 1.05668852197127 (1.01, 1.10) | 0.011 |
|  | Q3 (16,17) | 4283 | 85812/3234 | 1.12 (1.07, 1.17) | < 0.001 | 1.07125190725287 (1.03, 1.12) | 0.002 |
|  | Q4 (17,31.4) | 4519 | 85658/3248 | 1.19 (1.14, 1.24) | < 0.001 | 1.12919416123964 (1.08, 1.18) | < 0.001 |
|  | P for trend | - | - | - | < 0.001 | - | < 0.001 |
| NO_2_ | per 10 μg/m^3^ increment | 18891 | 372027/14192 | 1.15 (1.13, 1.17) | < 0.001 | 1.10524035489718 (1.08, 1.13) | < 0.001 |
|  | Q1 (12.9,21.5) | 4255 | 94875/3614 | ref | ref | ref | ref |
|  | Q2 (21.5,26.2) | 4876 | 93364/3570 | 1.18 (1.14, 1.23) | < 0.001 | 1.12262362489622 (1.08, 1.17) | < 0.001 |
|  | Q3 (26.2,31.3) | 4929 | 92676/3546 | 1.24 (1.19, 1.29) | < 0.001 | 1.15676978799126 (1.11, 1.21) | < 0.001 |
|  | Q4 (31.3,108) | 4831 | 91112/3461 | 1.35 (1.29, 1.40) | < 0.001 | 1.24013985709694 (1.19, 1.29) | < 0.001 |
|  | P for trend | - | - | - | < 0.001 | - | < 0.001 |
| NO_x_ | per 20 μg/m^3^ increment | 18891 | 372027/14192 | 1.13 (1.1147, 1.1524) | < 0.001 | 1.09277051172439 (1.07, 1.11) | < 0.001 |
|  | Q1 (19.7,34.4) | 4248 | 94859/3612 | ref | ref | ref | ref |
|  | Q2 (34.4,42.4) | 4782 | 93147/3553 | 1.17 (1.12, 1.22) | < 0.001 | 1.11304036772155 (1.07, 1.16) | < 0.001 |
|  | Q3 (42.4,50.8) | 4836 | 92743/3534 | 1.23 (1.18, 1.28) | < 0.001 | 1.1461860850974 (1.10, 1.19) | < 0.001 |
|  | Q4 (50.8,266) | 5025 | 91278/3493 | 1.38 (1.32, 1.43) | < 0.001 | 1.25467069597856 (1.20, 1.31) | < 0.001 |
|  | P for trend | - | - | - | < 0.001 | - | < 0.001 |

Model a: age (as the underlying time scale) and gender.

Model b: age (as the underlying time scale), sex, ethnicity, BMI, education, household income, smoking status, drinking status, diet score, physical activity, and residence location distance to the coast.

Abbreviations: UTI, urinary tract infection; CI, confidence interval; HR, hazard ratio; NO_2_, nitrogen dioxide; NO_x_, nitrogen oxide; PM_2.5_, particulate matter with aerodynamic diameters of ≤2.5 μm; PM_coarse_, particulate matter with aerodynamic diameters between 2.5 and 10 μm; PM_10_, particulate matter with aerodynamic diameters of ≤10 μm; Q, quartile.

**Table S4** Multivariable‑adjusted hazard ratios (95% CI) for air pollution concentrations with the risk of MRSI in the UK Biobank study, after excluding cases of MRSI that occurred within 3 years of follow-up.

| **Pollution** | **Increase** | **Events** | **Total/Person-years** | **Model a**  **HR (95% CI)** | **P** | **Model b**  **HR (95% CI)** | **P** |
| --- | --- | --- | --- | --- | --- | --- | --- |
| PM_2.5_ | per 5 μg/m^3^ increment | 2009 | 369077/15659 | 1.43 (1.17, 1.74) | < 0.001 | 1.32 (1.08, 1.62) | 0.007 |
|  | Q1 (8.17,9.29) | 463 | 93884/3991 | ref | ref | ref | ref |
|  | Q2 (9.29,9.93) | 519 | 92233/3905 | 1.16 (1.02, 1.32) | 0.019 | 1.14 (1.01, 1.30) | 0.036 |
|  | Q3 (9.93,10.6) | 521 | 92889/3933 | 1.18 (1.05, 1.34) | 0.008 | 1.15 (1.02, 1.31) | 0.025 |
|  | Q4 (10.6,21.3) | 506 | 90071/3830 | 1.23 (1.08, 1.40) | 0.001 | 1.18 (1.04, 1.34) | 0.011 |
|  | P for trend | - | - | - | 0.001 | - | 0.014 |
| PM_coarse_ | per 5 μg/m^3^ increment | 2009 | 369077/15659 | 1.16 (0.92, 1.47) | 0.206 | 1.132 (0.89, 1.44) | 0.305 |
|  | Q1 (5.57,5.84) | 458 | 94214/4022 | ref | ref | ref | ref |
|  | Q2 (5.84,6.11) | 514 | 92747/3939 | 1.14 (1.01, 1.30) | 0.037 | 1.13 (1.00, 1.28) | 0.053 |
|  | Q3 (6.11,6.64) | 518 | 91052/3836 | 1.19 (1.05, 1.35) | 0.006 | 1.17 (1.03, 1.33) | 0.013 |
|  | Q4 (6.64,12.8) | 519 | 91064/3861 | 1.19 (1.05, 1.35) | 0.007 | 1.16 (1.03, 1.32) | 0.019 |
|  | P for trend | - | - | - | 0.029 | - | 0.067 |
| PM_10_ | per 10 μg/m^3^ increment | 2009 | 369077/15659 | 1.32 (1.06, 1.66) | 0.014 | 1.26 (1.00, 1.58) | 0.046 |
|  | Q1 (11.8,15.2) | 474 | 93142/3967 | ref | ref | ref | ref |
|  | Q2 (15.2,16) | 494 | 93165/3969 | 1.06 (0.94, 1.21) | 0.341 | 1.05 (0.93, 1.19) | 0.448 |
|  | Q3 (16,17) | 530 | 91368/3854 | 1.18 (1.05, 1.34) | 0.007 | 1.16 (1.02, 1.31) | 0.021 |
|  | Q4 (17,31.4) | 511 | 91402/3869 | 1.14 (1.01, 1.30) | 0.035 | 1.11 (0.98, 1.26) | 0.094 |
|  | P for trend | - | - | - | 0.021 | - | 0.066 |
| NO_2_ | per 10 μg/m^3^ increment | 2118 | 396423/16885 | 1.13 (1.07, 1.19) | < 0.001 | 1.11 (1.05, 1.17) | < 0.001 |
|  | Q1 (12.9,21.5) | 461 | 100475/4293 | ref | ref | ref | ref |
|  | Q2 (21.5,26.2) | 552 | 99593/4247 | 1.22 (1.08, 1.39) | 0.001 | 1.20 (1.06, 1.36) | 0.003 |
|  | Q3 (26.2,31.3) | 548 | 99101/4224 | 1.26 (1.11, 1.42) | < 0.001 | 1.22 (1.08, 1.39) | 0.001 |
|  | Q4 (31.3,108) | 557 | 97254/4122 | 1.35 (1.19, 1.53) | < 0.001 | 1.29 (1.14, 1.47) | < 0.001 |
|  | P for trend | - | - | - | < 0.001 | - | < 0.001 |
| NO_x_ | per 20 μg/m^3^ increment | 2118 | 396423/16885 | 1.09 (1.04, 1.15) | < 0.001 | 1.07 (1.02, 1.13) | 0.006 |
|  | Q1 (19.7,34.4) | 477 | 100380/4287 | ref | ref | ref | ref |
|  | Q2 (34.4,42.4) | 536 | 99367/4231 | 1.15 (1.02, 1.30) | 0.024 | 1.13 (1.00, 1.28) | 0.050 |
|  | Q3 (42.4,50.8) | 553 | 98972/4208 | 1.23 (1.09, 1.39) | < 0.001 | 1.19 (1.06, 1.35) | 0.005 |
|  | Q4 (50.8,266) | 552 | 97704/4159 | 1.29 (1.14, 1.45) | < 0.001 | 1.23 (1.09, 1.39) | 0.001 |
|  | P for trend | - | - | - | < 0.001 | - | 0.001 |

Model a: age (as the underlying time scale) and gender.

Model b: age (as the underlying time scale), sex, ethnicity, BMI, education, household income, smoking status, drinking status, diet score, physical activity, and residence location distance to the coast.

Abbreviations: MRSI, male reproductive system infection; CI, confidence interval; HR, hazard ratio; NO_2_, nitrogen dioxide; NO_x_, nitrogen oxide; PM_2.5_, particulate matter with aerodynamic diameters of ≤2.5 μm; PM_coarse_, particulate matter with aerodynamic diameters between 2.5 and 10 μm; PM_10_, particulate matter with aerodynamic diameters of ≤10 μm; Q, quartile.

**Table S5** Multivariable‑adjusted hazard ratios (95% CI) for air pollution concentrations with the risk of UTI in the UK Biobank study, after excluding participants who lived in their current residence for less than 5 years.

| **Pollution** | **Increase** | **Events** | **Total/Person-years** | **Model a**  **HR (95% CI)** | **P** | **Model b**  **HR (95% CI)** | **P** |
| --- | --- | --- | --- | --- | --- | --- | --- |
| PM_2.5_ | per 5 μg/m^3^ increment | 17384 | 294583/11108 | 1.68 (1.58, 1.80) | < 0.001 | 1.43 (1.33, 1.53) | < 0.001 |
|  | Q1 (8.17,9.29) | 4536 | 69872/2654 | ref | ref | ref | ref |
|  | Q2 (9.29,9.93) | 4262 | 74905/2815 | 0.82 (0.79, 0.86) | < 0.001 | 0.87 (0.83, 0.91) | < 0.001 |
|  | Q3 (9.93,10.6) | 4520 | 74441/2802 | 0.90 (0.87, 0.94) | < 0.001 | 0.92 (0.89, 0.96) | < 0.001 |
|  | Q4 (10.6,21.3) | 4066 | 75365/2837 | 0.76 (0.73, 0.79) | < 0.001 | 0.84 (0.80, 0.87) | < 0.001 |
|  | P for trend | - | - | - | < 0.001 | - | < 0.001 |
| PM_coarse_ | per 5 μg/m^3^ increment | 17384 | 294583/11108 | 1.24 (1.15, 1.34) | < 0.001 | 1.18 (1.09, 1.27) | < 0.001 |
|  | Q1 (5.57,5.84) | 4320 | 74596/2815 | ref | ref | ref | ref |
|  | Q2 (5.84,6.11) | 4349 | 72475/2711 | 1.06 (1.02, 1.11) | < 0.001 | 1.05 (1.01, 1.09) | 0.028 |
|  | Q3 (6.11,6.64) | 4487 | 71769/2711 | 1.10 (1.05, 1.15) | < 0.001 | 1.08 (1.04, 1.13) | < 0.001 |
|  | Q4 (6.64,12.8) | 4228 | 75743/2871 | 0.95 (0.91, 0.99) | 0.014 | 0.97 (0.93, 1.01) | 0.131 |
|  | P for trend | - | - | - | < 0.001 | - | < 0.001 |
| PM_10_ | per 10 μg/m^3^ increment | 17384 | 294583/11108 | 1.37 (1.27, 1.49) | < 0.001 | 1.24 (1.15, 1.34) | < 0.001 |
|  | Q1 (11.8,15.2) | 4465 | 75142/2849 | ref | ref | ref | ref |
|  | Q2 (15.2,16) | 4322 | 73296/2743 | 1.03 (0.99, 1.07) | 0.175 | 1.02 (0.97, 1.06) | 0.439 |
|  | Q3 (16,17) | 4465 | 71911/2711 | 1.09 (1.04, 1.13) | < 0.001 | 1.07 (1.02, 1.11) | 0.002 |
|  | Q4 (17,31.4) | 4132 | 74234/2805 | 0.93 (0.89, 0.97) | < 0.001 | 0.96 (0.92, 1.00) | 0.038 |
|  | P for trend | - | - | - | < 0.001 | - | < 0.001 |
| NO_2_ | per 10 μg/m^3^ increment | 18881 | 316329/11991 | 1.14 (1.12, 1.16) | < 0.001 | 1.10 (1.08, 1.12) | < 0.001 |
|  | Q1 (12.9,21.5) | 4906 | 80402/3055 | ref | ref | ref | ref |
|  | Q2 (21.5,26.2) | 4967 | 79780/3032 | 1.05 (1.01, 1.09) | 0.026 | 1.03 (0.99, 1.07) | 0.166 |
|  | Q3 (26.2,31.3) | 4740 | 75961/2867 | 1.13 (1.08, 1.17) | < 0.001 | 1.09 (1.05, 1.14) | < 0.001 |
|  | Q4 (31.3,108) | 4268 | 80186/3038 | 0.86 (0.82, 0.90) | < 0.001 | 0.90 (0.87, 0.94) | < 0.001 |
|  | P for trend | - | - | - | < 0.001 | - | < 0.001 |
| NO_x_ | per 20 μg/m^3^ increment | 18881 | 316329/11991 | 1.13 (1.11, 1.15) | < 0.001 | 1.09 (1.07, 1.11) | < 0.001 |
|  | Q1 (19.7,34.4) | 4817 | 80187/3039 | ref | ref | ref | ref |
|  | Q2 (34.4,42.4) | 4860 | 79724/3019 | 1.05 (1.01, 1.09) | 0.016 | 1.02 (0.98, 1.07) | 0.242 |
|  | Q3 (42.4,50.8) | 4918 | 76102/2892 | 1.17 (1.12, 1.22) | < 0.001 | 1.12 (1.07, 1.16) | < 0.001 |
|  | Q4 (50.8,266) | 4286 | 80316/3040 | 0.87 (0.84, 0.91) | < 0.001 | 0.91 (0.88, 0.95) | < 0.001 |
|  | P for trend | - | - | - | < 0.001 | - | < 0.001 |

Model a: age (as the underlying time scale) and gender.

Model b: age (as the underlying time scale), sex, ethnicity, BMI, education, household income, smoking status, drinking status, diet score, physical activity, and residence location distance to the coast.

Abbreviations: UTI, urinary tract infection; CI, confidence interval; HR, hazard ratio; NO_2_, nitrogen dioxide; NO_x_, nitrogen oxide; PM_2.5_, particulate matter with aerodynamic diameters of ≤2.5 μm; PM_coarse_, particulate matter with aerodynamic diameters between 2.5 and 10 μm; PM_10_, particulate matter with aerodynamic diameters of ≤10 μm; Q, quartile.

**Table S6** Multivariable‑adjusted hazard ratios (95% CI) for air pollution concentrations with the risk of MRSI in the UK Biobank study, after excluding participants who lived in their current residence for less than 5 years.

| **Pollution** | **Increase** | **Events** | **Total/Person-years** | **Model a** | **P** | **Model b** | **P** |
| --- | --- | --- | --- | --- | --- | --- | --- |
| PM_2.5_ | per 5 μg/m^3^ increment | 2133 | 313967/11439 | 1.29 (1.06, 1.58) | 0.011 | 1.22 (1.00, 1.49) | 0.054 |
|  | Q1 (8.17,9.29) | 506 | 74989/2741 | ref | ref | ref | ref |
|  | Q2 (9.29,9.93) | 577 | 79595/2894 | 1.01 (0.90, 1.14) | 0.796 | 1.03 (0.92, 1.17) | 0.562 |
|  | Q3 (9.93,10.6) | 550 | 79539/2892 | 0.99 (0.88, 1.12) | 0.904 | 1.00 (0.89, 1.13) | 0.948 |
|  | Q4 (10.6,21.3) | 500 | 79844/2912 | 0.86 (0.76, 0.97) | 0.016 | 0.89 (0.78, 1.01) | 0.062 |
|  | P for trend | - | - | - | 0.025 | - | 0.096 |
| PM_coarse_ | per 5 μg/m^3^ increment | 2133 | 313967/11439 | 1.18 (0.94, 1.49) | 0.149 | 1.16 (0.92, 1.46) | 0.201 |
|  | Q1 (5.57,5.84) | 553 | 79378/2896 | ref | ref | ref | ref |
|  | Q2 (5.84,6.11) | 539 | 77276/2792 | 1.03 (0.91, 1.16) | 0.631 | 1.02 (0.91, 1.15) | 0.685 |
|  | Q3 (6.11,6.64) | 552 | 76737/2795 | 1.05 (0.93, 1.18) | 0.423 | 1.04 (0.93, 1.17) | 0.497 |
|  | Q4 (6.64,12.8) | 489 | 80576/2956 | 0.86 (0.77, 0.98) | 0.020 | 0.87 (0.77, 0.98) | 0.027 |
|  | P for trend | - | - | - | 0.011 | - | 0.021 |
| PM_10_ | per 10 μg/m^3^ increment | 2133 | 313967/11439 | 1.32 (1.06, 1.64) | 0.013 | 1.27 (1.02, 1.59) | 0.033 |
|  | Q1 (11.8,15.2) | 533 | 80148/2935 | ref | ref | ref | ref |
|  | Q2 (15.2,16) | 549 | 78092/2825 | 1.09 (0.97, 1.23) | 0.148 | 1.08 (0.96, 1.22) | 0.186 |
|  | Q3 (16,17) | 548 | 76850/2794 | 1.11 (0.98, 1.25) | 0.093 | 1.10 (0.97, 1.24) | 0.125 |
|  | Q4 (17,31.4) | 503 | 78877/2885 | 0.94 (0.84, 1.07) | 0.348 | 0.95 (0.84, 1.08) | 0.443 |
|  | P for trend | - | - | - | 0.005 | - | 0.015 |
| NO_2_ | per 10 μg/m^3^ increment | 2244 | 337454/12354 | 1.11 (1.05, 1.17) | < 0.001 | 1.09 (1.04, 1.16) | 0.001 |
|  | Q1 (12.9,21.5) | 607 | 85823/3147 | ref | ref | ref | ref |
|  | Q2 (21.5,26.2) | 570 | 85370/3128 | 0.97 (0.87, 1.09) | 0.634 | 0.97 (0.86, 1.08) | 0.548 |
|  | Q3 (26.2,31.3) | 569 | 81248/2957 | 1.07 (0.95, 1.20) | 0.242 | 1.05 (0.94, 1.18) | 0.405 |
|  | Q4 (31.3,108) | 498 | 85013/3121 | 0.82 (0.72, 0.92) | < 0.001 | 0.83 (0.74, 0.93) | 0.002 |
|  | P for trend | - | - | - | < 0.001 | - | < 0.001 |
| NO_x_ | per 20 μg/m^3^ increment | 2244 | 337454/12354 | 1.09 (1.03, 1.14) | 0.001 | 1.07 (1.02, 1.13) | 0.011 |
|  | Q1 (19.7,34.4) | 574 | 85592/3133 | ref | ref | ref | ref |
|  | Q2 (34.4,42.4) | 602 | 85150/3112 | 1.09 (0.97, 1.22) | 0.148 | 1.08 (0.96, 1.21) | 0.195 |
|  | Q3 (42.4,50.8) | 550 | 81616/2987 | 1.08 (0.96, 1.21) | 0.224 | 1.05 (0.94, 1.19) | 0.385 |
|  | Q4 (50.8,266) | 518 | 85096/3122 | 0.89 (0.79, 1.00) | 0.057 | 0.91 (0.80, 1.02) | 0.102 |
|  | P for trend | - | - | - | 0.001 | - | 0.009 |

Model a: age (as the underlying time scale) and gender.

Model b: age (as the underlying time scale), sex, ethnicity, BMI, education, household income, smoking status, drinking status, diet score, physical activity, and residence location distance to the coast.

Abbreviations: MRSI, male reproductive system infection; CI, confidence interval; HR, hazard ratio; NO_2_, nitrogen dioxide; NO_x_, nitrogen oxide; PM_2.5_, particulate matter with aerodynamic diameters of ≤2.5 μm; PM_coarse_, particulate matter with aerodynamic diameters between 2.5 and 10 μm; PM_10_, particulate matter with aerodynamic diameters of ≤10 μm; Q, quartile.

**Table S7** Multivariable‑adjusted hazard ratios (95% CI) for air pollution concentrations with the risk of UTI in the UK Biobank study, after excluding extreme values of PM_2.5_, PM_coarse_, PM_10_, NO_2_, and NO_x_ (percentiles outside 1–99).

| Pollution | Increase | Events | Total/Person-years | Model a  HR (95% CI) | P | Model b  HR (95% CI) | P |
| --- | --- | --- | --- | --- | --- | --- | --- |
| PM_2.5_ | per 5 μg/m^3^ increment | 19404 | 333320/12571 | 1.65 (1.55, 1.77) | < 0.001 | 1.40 (1.31, 1.49) | < 0.001 |
|  | Q1 (8.17,9.29) | 5127 | 80942/3071 | ref | ref | ref | ref |
|  | Q2 (9.29,9.93) | 4725 | 83387/3135 | 0.83 (0.80, 0.86) | < 0.001 | 0.88 (0.84, 0.91) | < 0.001 |
|  | Q3 (9.93,10.6) | 5027 | 83779/3154 | 0.90 (0.87, 0.94) | < 0.001 | 0.93 (0.89, 0.97) | < 0.001 |
|  | Q4 (10.6,21.3) | 4525 | 85212/3210 | 0.76 (0.73, 0.79) | < 0.001 | 0.84 (0.80, 0.87) | < 0.001 |
|  | P for trend | - | - | - | < 0.001 | - | < 0.001 |
| PM_coarse_ | per 5 μg/m^3^ increment | 19404 | 333320/12571 | 1.22 (1.13, 1.32) | < 0.001 | 1.15 (1.07, 1.25) | < 0.001 |
|  | Q1 (5.57,5.84) | 4823 | 83820/3164 | ref | ref | ref | ref |
|  | Q2 (5.84,6.11) | 4891 | 82175/3075 | 1.06 (1.02, 1.10) | 0.003 | 1.05 (1.01, 1.09) | 0.018 |
|  | Q3 (6.11,6.64) | 5011 | 82087/3099 | 1.08 (1.04, 1.13) | < 0.001 | 1.06 (1.02, 1.11) | 0.002 |
|  | Q4 (6.64,12.8) | 4679 | 85238/3232 | 0.93 (0.90, 0.97) | 0.001 | 0.96 (0.92, 0.99) | 0.026 |
|  | P for trend | - | - | - | < 0.001 | - | < 0.001 |
| PM_10_ | per 10 μg/m^3^ increment | 19404 | 333320/12571 | 1.35 (1.26, 1.46) | < 0.001 | 1.22 (1.13, 1.31) | < 0.001 |
|  | Q1 (11.8,15.2) | 4961 | 84025/3187 | ref | ref | ref | ref |
|  | Q2 (15.2,16) | 4827 | 82540/3090 | 1.03 (0.99, 1.07) | 0.109 | 1.02 (0.98, 1.06) | 0.333 |
|  | Q3 (16,17) | 4987 | 82446/3106 | 1.07 (1.03, 1.12) | < 0.001 | 1.05 (1.01, 1.09) | 0.011 |
|  | Q4 (17,31.4) | 4629 | 84309/3187 | 0.92 (0.88, 0.96) | < 0.001 | 0.95 (0.91, 0.99) | 0.016 |
|  | P for trend | - | - | - | < 0.001 | - | < 0.001 |
| NO_2_ | per 10 μg/m^3^ increment | 21048 | 357705/13561 | 1.14 (1.12, 1.16) | < 0.001 | 1.09 (1.07, 1.11) | < 0.001 |
|  | Q1 (12.9,21.5) | 5413 | 89698/3410 | ref | ref | ref | ref |
|  | Q2 (21.5,26.2) | 5483 | 89204/3389 | 1.05 (1.01, 1.09) | 0.013 | 1.03 (0.99, 1.07) | 0.14 |
|  | Q3 (26.2,31.3) | 5360 | 87649/3307 | 1.13 (1.09, 1.17) | < 0.001 | 1.09 (1.05, 1.14) | < 0.001 |
|  | Q4 (31.3,108) | 4792 | 91154/3455 | 0.86 (0.83, 0.89) | < 0.001 | 0.90 (0.87, 0.94) | < 0.001 |
|  | P for trend | - | - | - | < 0.001 | - | < 0.001 |
| NO_x_ | per 20 μg/m^3^ increment | 21048 | 357705/13561 | 1.12 (1.11, 1.14) | < 0.001 | 1.08 (1.07, 1.10) | < 0.001 |
|  | Q1 (19.7,34.4) | 5321 | 89530/3394 | ref | ref | ref | ref |
|  | Q2 (34.4,42.4) | 5363 | 89193/3377 | 1.05 (1.01, 1.09) | 0.013 | 1.02 (0.98, 1.06) | 0.249 |
|  | Q3 (42.4,50.8) | 5570 | 87880/3339 | 1.17 (1.13, 1.21) | < 0.001 | 1.12 (1.07, 1.16) | < 0.001 |
|  | Q4 (50.8,266) | 4794 | 91102/3451 | 0.87 (0.84, 0.91) | < 0.001 | 0.91 (0.88, 0.95) | < 0.001 |
|  | P for trend | - | - | - | < 0.001 | - | < 0.001 |

Model a: age (as the underlying time scale) and gender.

Model b: age (as the underlying time scale), sex, ethnicity, BMI, education, household income, smoking status, drinking status, diet score, physical activity, and residence location distance to the coast.

Abbreviations: UTI, urinary tract infection; CI, confidence interval; HR, hazard ratio; NO_2_, nitrogen dioxide; NO_x_, nitrogen oxide; PM_2.5_, particulate matter with aerodynamic diameters of ≤2.5 μm; PM_coarse_, particulate matter with aerodynamic diameters between 2.5 and 10 μm; PM_10_, particulate matter with aerodynamic diameters of ≤10 μm; Q, quartile.

**Table S8** Multivariable‑adjusted hazard ratios (95% CI) for air pollution concentrations with the risk of MRSI in the UK Biobank study, after excluding extreme values of PM_2.5_, PM_coarse_, PM_10_, NO_2_, and NO_x_ (percentiles outside 1–99).

| **Pollution** | **Increase** | **Events** | **Total/Person-years** | **Model a**  **HR (95% CI)** | **P** | **Model b**  **HR (95% CI)** | **P** |
| --- | --- | --- | --- | --- | --- | --- | --- |
| PM_2.5_ | per 5 μg/m^3^ increment | 2396 | 355553/12963 | 1.39 (1.15, 1.67) | < 0.001 | 1.30 (1.08, 1.57) | 0.006 |
|  | Q1 (8.17,9.29) | 590 | 86879/3176 | ref | ref | ref | ref |
|  | Q2 (9.29,9.93) | 618 | 88755/3229 | 0.98 (0.87, 1.09) | 0.663 | 1.00 (0.89, 1.12) | 0.982 |
|  | Q3 (9.93,10.6) | 638 | 89562/3258 | 1.02 (0.91, 1.14) | 0.735 | 1.03 (0.92, 1.16) | 0.550 |
|  | Q4 (10.6,21.3) | 550 | 90357/3300 | 0.83 (0.74, 0.94) | 0.002 | 0.87 (0.77, 0.97) | 0.016 |
|  | P for trend | - | - | - | 0.002 | - | 0.015 |
| PM_coarse_ | per 5 μg/m^3^ increment | 2396 | 355553/12963 | 1.12 (0.91, 1.40) | 0.290 | 1.09 (0.88, 1.36) | 0.410 |
|  | Q1 (5.57,5.84) | 612 | 89333/3262 | ref | ref | ref | ref |
|  | Q2 (5.84,6.11) | 617 | 87718/3172 | 1.06 (0.94, 1.18) | 0.340 | 1.05 (0.94, 1.17) | 0.416 |
|  | Q3 (6.11,6.64) | 617 | 87776/3198 | 1.04 (0.93, 1.16) | 0.491 | 1.03 (0.92, 1.15) | 0.620 |
|  | Q4 (6.64,12.8) | 550 | 90726/3331 | 0.88 (0.78, 0.98) | 0.026 | 0.88 (0.79, 0.99) | 0.038 |
|  | P for trend | - | - | - | 0.019 | - | 0.045 |
| PM_10_ | per 10 μg/m^3^ increment | 2396 | 355553/12963 | 1.30 (1.06, 1.60) | 0.012 | 1.24 (1.01, 1.53) | 0.041 |
|  | Q1 (11.8,15.2) | 592 | 89743/3288 | ref | ref | ref | ref |
|  | Q2 (15.2,16) | 624 | 88047/3188 | 1.11 (0.99, 1.24) | 0.069 | 1.10 (0.98, 1.23) | 0.103 |
|  | Q3 (16,17) | 613 | 88108/3204 | 1.09 (0.97, 1.22) | 0.154 | 1.07 (0.96, 1.20) | 0.234 |
|  | Q4 (17,31.4) | 567 | 89655/3283 | 0.94 (0.84, 1.06) | 0.317 | 0.95 (0.85, 1.07) | 0.420 |
|  | P for trend | - | - | - | 0.009 | - | 0.031 |
| NO_2_ | per 10 μg/m^3^ increment | 2524 | 381895/13990 | 1.14 (1.09, 1.20) | < 0.001 | 1.12 (1.07, 1.18) | < 0.001 |
|  | Q1 (12.9,21.5) | 656 | 95883/3518 | ref | ref | ref | ref |
|  | Q2 (21.5,26.2) | 647 | 95538/3502 | 1.02 (0.92, 1.14) | 0.706 | 1.01 (0.91, 1.13) | 0.835 |
|  | Q3 (26.2,31.3) | 673 | 93754/3415 | 1.13 (1.01, 1.26) | 0.027 | 1.10 (0.99, 1.23) | 0.082 |
|  | Q4 (31.3,108) | 548 | 96720/3555 | 0.82 (0.73, 0.91) | < 0.001 | 0.83 (0.74, 0.93) | 0.001 |
|  | P for trend | - | - | - | < 0.001 | - | < 0.001 |
| NO_x_ | per 20 μg/m^3^ increment | 2524 | 381895/13990 | 1.10 (1.05, 1.15) | < 0.001 | 1.08 (1.03, 1.14) | < 0.001 |
|  | Q1 (19.7,34.4) | 634 | 95665/3503 | ref | ref | ref | ref |
|  | Q2 (34.4,42.4) | 674 | 95347/3487 | 1.10 (0.99, 1.23) | 0.084 | 1.09 (0.98, 1.21) | 0.123 |
|  | Q3 (42.4,50.8) | 645 | 94261/3451 | 1.10 (0.99, 1.23) | 0.090 | 1.07 (0.96, 1.20) | 0.218 |
|  | Q4 (50.8,266) | 571 | 96622/3549 | 0.88 (0.78, 0.98) | 0.022 | 0.89 (0.80, 1.00) | 0.047 |
|  | P for trend | - | - | - | < 0.001 | - | < 0.001 |

Model a: age (as the underlying time scale) and gender.

Model b: age (as the underlying time scale), sex, ethnicity, BMI, education, household income, smoking status, drinking status, diet score, physical activity, and residence location distance to the coast.

Abbreviations: MRSI, male reproductive system infection; CI, confidence interval; HR, hazard ratio; NO_2_, nitrogen dioxide; NO_x_, nitrogen oxide; PM_2.5_, particulate matter with aerodynamic diameters of ≤2.5 μm; PM_coarse_, particulate matter with aerodynamic diameters between 2.5 and 10 μm; PM_10_, particulate matter with aerodynamic diameters of ≤10 μm; Q, quartile.

**Supporting Figure 1**. Stratification analyses of the associations between long-term exposure to PM_2.5_ and the risk of UTIs.

Adjusted for age, gender, ethnicity, BMI, education, smoking, physical activity, drinking, healthy diet score, household income and residence location distance to the coast.

Abbreviations: BMI, body mass index; CI, confidence interval; HR, hazards ratio; PM_2.5_, particulate matter with aerodynamic diameter ≤2.5 μm.

**Supporting Figure 2**. Stratification analyses of the associations between long-term exposure to PM_coarse_ and the risk of UTIs.

Adjusted for age, gender, ethnicity, BMI, education, smoking, physical activity, drinking, healthy diet score, household income and residence location distance to the coast.

Abbreviations: BMI, body mass index; CI, confidence interval; HR, hazards ratio; PM_coarse_, particulate matter with an aerodynamic diameter between 2.5 and 10 μm.

**Supporting Figure 3**. Stratification analyses of the associations between long-term exposure to PM_10_ and the risk of UTIs.

Adjusted for age, gender, ethnicity, BMI, education, smoking, physical activity, drinking, healthy diet score, household income and residence location distance to the coast.

Abbreviations: BMI, body mass index; CI, confidence interval; HR, hazards ratio; PM_10_, particulate matter with an aerodynamic diameter ≤10 μm.

**Supporting Figure 4**. Stratification analyses of the associations between long-term exposure to NO_2_ and the risk of UTIs.

Adjusted for age, gender, ethnicity, BMI, education, smoking, physical activity, drinking, healthy diet score, household income and residence location distance to the coast.

Abbreviations: BMI, body mass index; CI, confidence interval; HR, hazards ratio; NO_2_, nitrogen dioxide.

**Supporting Figure 5.** Stratification analyses of the associations between long-term exposure to NO_x_ and the risk of UTIs.

Adjusted for age, gender, ethnicity, BMI, education, smoking, physical activity, drinking, healthy diet score, household income and residence location distance to the coast.

Abbreviations: BMI, body mass index; CI, confidence interval; HR, hazards ratio; NO_x_, nitrogen oxide.

**Supporting Figure 6**. Stratification analyses of the associations between long-term exposure to PM_2.5_ and the risk of MRSIs.

Adjusted for age, gender, ethnicity, BMI, education, smoking, physical activity, drinking, healthy diet score, household income and residence location distance to the coast.

Abbreviations: BMI, body mass index; CI, confidence interval; HR, hazards ratio; PM_2.5_, particulate matter with aerodynamic diameter ≤2.5 μm.

**Supporting Figure 7**. Stratification analyses of the associations between long-term exposure to PM_coarse_ and the risk of MRSIs.

Adjusted for age, gender, ethnicity, BMI, education, smoking, physical activity, drinking, healthy diet score, household income and residence location distance to the coast.

Abbreviations: BMI, body mass index; CI, confidence interval; HR, hazards ratio; PM_coarse_, particulate matter with an aerodynamic diameter between 2.5 and 10 μm.

**Supporting Figure 8**. Stratification analyses of the associations between long-term exposure to PM_10_ and the risk of MRSIs.

Adjusted for age, gender, ethnicity, BMI, education, smoking, physical activity, drinking, healthy diet score, household income and residence location distance to the coast.

Abbreviations: BMI, body mass index; CI, confidence interval; HR, hazards ratio; PM_10_, particulate matter with an aerodynamic diameter ≤10 μm.

**Supporting Figure 9**. Stratification analyses of the associations between long-term exposure to NO_2_ and the risk of MRSIs.

Adjusted for age, gender, ethnicity, BMI, education, smoking, physical activity, drinking, healthy diet score, household income and residence location distance to the coast.

Abbreviations: BMI, body mass index; CI, confidence interval; HR, hazards ratio; NO_2_, nitrogen dioxide.

**Supporting Figure 10.** Stratification analyses of the associations between long-term exposure to NO_x_ and the risk of MRSIs.

Adjusted for age, gender, ethnicity, BMI, education, smoking, physical activity, drinking, healthy diet score, household income and residence location distance to the coast.

Abbreviations: BMI, body mass index; CI, confidence interval; HR, hazards ratio; NO_x_, nitrogen oxide.
